# Gluten Immunogenic Peptides as Standard for the Evaluation of Potential Harmful Prolamin Content in Food and Human Specimen

**DOI:** 10.3390/nu10121927

**Published:** 2018-12-05

**Authors:** Ángel Cebolla, María de Lourdes Moreno, Laura Coto, Carolina Sousa

**Affiliations:** 1 Biomedal Ltd., 41900 Sevilla, Spain; acebolla@biomedal.com (Á.C.); laura.coto@biomedal.com (L.C.); 2 Facultad de Farmacia, Departamento de Microbiología y Parasitología, Universidad de Sevilla, 41012 Sevilla, Spain; lmoreno@us.es

**Keywords:** gluten immunogenic peptides, celiac disease, gluten quantitation, gluten food analysis

## Abstract

Gluten is a complex mixture of storage proteins in cereals like wheat, barley, and rye. Prolamins are the main components of gluten. Their high content in proline and glutamine makes them water-insoluble and difficult to digest in the gastrointestinal tract. Partial digestion generates peptide sequences which trigger immune responses in celiac and gluten-sensitive patients. Gluten detection in food is challenging because of the diversity, in various food matrices, of protein proportions or modifications and the huge number of immunogenic sequences with differential potential immunoactivity. Attempts to develop standard reference materials have been unsuccessful. Recent studies have reported the detection of a limited number of dominant Gluten Immunogenic Peptides (GIP) that share similarities to epitopes presented in the *α*-gliadin 33-mer, which showed to be highly proteolytic resistant and is considered to be the most immunodominant peptide within gluten in celiac disease (CD). GIP were detectable and quantifiable in very different kind of difficult to analyze food, revealing the potential immunogenicity by detecting T-cell activity of celiac patients. But GIP were also found in stool and urine of celiac patients on a supposedly gluten-free diet (GFD), showing the capacity to resist and be absorbed and excreted from the body, providing the first simple and objective means to assess adherence to the GFD. Methods to specifically and sensitively detect the most active GIP in food and biological fluids are rational candidates may use similar analytical standard references for determination of the immunopathological risk of gluten exposure in gluten-related diseases.

## 1. Introduction

**Figure 1:**
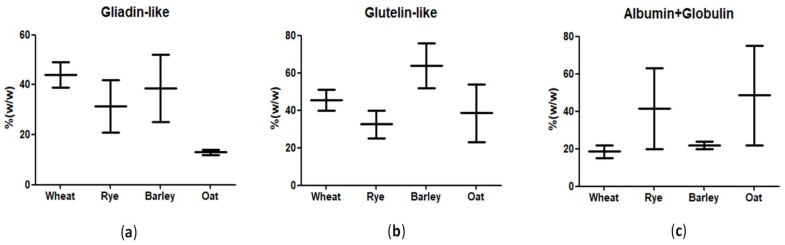
Relative content in gluten proteins of the most consumed cereals expressed as medium average content found in literature (%). (**a**) Gliadin-like proteins; (**b**) Glutelin-like proteins; (**c**) Albumin + Globulin proteins [8,9,10,11,12].

Gluten is a complex mixture of storage proteins found in cereal seeds composed of hundreds of related but distinct proteins, mainly prolamins and glutelins [1]. Prolamins contribute to the cohesiveness and extensibility of the gluten, whereas glutelins play a role in the maintenance of the elasticity and strength of the gluten [2]. Prolamins have been widely studied due to their contribution to the quality of the end product of bakery and pasta foods, including the rheological characteristics of dough made from wheat flour [3,4]. Prolamins are the major storage proteins in wheat (gliadin), barley (hordein), rye (secalin), corn (zein), sorghum (kafirin), and a minor protein in oats (avenin) and rice (orzein) [5]. Oats species have the lowest variability in prolamin content, whereas wheats have the highest content (Figure 1). Glutelins are heterogeneous and can be separated using electrophoresis into over a dozen fractions of different molecular weights: high molecular weight (HMW) and low molecular weight (LMW) that in wheat are defined as glutenins. An appropriate percentage of HMW glutenins in wheat flour are responsible for the baking value of the flour [6]. Wheat proteins have been classically defined according to their solubility [3]. In wheat, they are categorized as albumins (soluble in water), globulins (soluble in saline solutions), gliadins (soluble in 60–70% ethanol), and glutenins (only soluble under stronger conditions, i.e., acids, reducing agents and detergents, urea, etc.) [7]. According to their electrophoretic mobility, gliadins are subdivided into three main groups: the first *α*-and *β*-gliadins, the second *ϒ*-gliadins and the third consists of *Ω*-gliadins [3].

High densities of proline and glutamine residues are present in the prolamins which are synthesized and deposited in the endosperm of the grain as primary source of nitrogen for protein synthesis, which occurs later during germination [13,14,15]. The proteins and polypeptides within the Prolamin Superfamily possess similar structures: signal peptide for translocation into cellular compartments, a non-repetitive N-terminal region, a non-repetitive C-terminal region and a long repetitive central region. The central region contains glutamine rich and proline-rich repeat units unique to each group [15]. However, there are differences in the number, content and properties of prolamin polypeptides among these cereals. Strictly speaking, the term “gluten” could comprise all prolamins including those of rice or maize. However, for simplification and practical reasons, the term gluten is typically restricted for prolamins with the ability to trigger immunotoxicity in certain groups of patients with gluten-sensitive spectrum, which includes three main forms: allergic (wheat allergy), autoimmune (celiac disease, (CD), dermatitis herpetiformis, and gluten ataxia), and immune-mediated (non-celiac gluten-sensitivity). This simplification often leads to confusion when determining the gluten content in different cereals by different immunologic methods. To understand the factors affecting gluten content estimations, we evaluated the recent literature on methodologies for detecting gluten, as well as the reference material for the relevant articles. The aim of this review article is to provide an overview of the current definition of gluten according to the latest methods and scientific understanding, and to discuss the appropriate standards to assess immunogenic gluten in foods and human biological samples.

The search was conducted in PubMed MEDLINE and SCOPUS databases. The following search terms were used: “gluten content”, “gluten analysis”, “celiac immunogenic peptides”, “gluten immunogenic peptides”, and “toxic gluten”. The keywords “toxicity and celiac disease”, “immunogenicity and celiac disease”, “harmful prolamin and celiac disease”, “gluten proteins and celiac disease”, “gluten immunogenic peptides and celiac disease”, and “gluten peptides and celiac disease” were also used.

## 2. Gluten as the Trigger for Autoimmunity

Gluten proteins found in wheat, barley, and rye are among the only food ingredients known to trigger an autoimmune condition: CD [16]. Exposure to gluten in combination to genetic predisposition and other unknown immunologic and environmental factors can trigger CD [17]. The main pathological lesion produced by gluten in CD is located in the proximal small intestine; however, the disease is increasingly considered a systemic disorder rather than a disease limited to the gastrointestinal tract. This is primarily explained by the fact that CD belongs to the group of autoimmune diseases (AD). In the full-blown lesion, there is loss of small bowel intestinal villi and infiltration of leukocytes, both in the epithelium and in the lamina propria. The chronic inflammatory condition of CD can be reversed with a gluten-free diet (GFD) [18]. The symptoms associated with gluten intake in CD can be diverse and include diarrhea, constipation, anemia, osteoporosis, dermatitis herpetiformis, fatigue, and infertility [19]. Moreover, a significantly increased prevalence of other AD has been reported in individuals with CD and their first-degree relatives as compared to controls, with an estimated burden of AD in CD cases up to 15% [20,21]. It has been suggested that these associations among CD and other AD may be explained by the sharing of a common pathogenic basis involving genetic susceptibility, similar environmental triggers, and the loss of intestinal barrier secondary to dysfunction of intercellular tight junctions with increased intestinal permeability, and possibly by other undiscovered mechanisms [20,21]. The type of symptoms and their intensity is largely dependent on the individual, and likely related to the pattern and amount of gluten consumption [22]. The HLA-DQ2 and HLA-DQ8 genotypes determine the risk of disease development because the corresponding encoded HLA-DQ2 and HLA-DQ8 bind to gluten peptides and present them to the T lymphocytes [23]. 

After ingestion of gluten-containing food, gluten is partially digested by enzymes of the gastrointestinal track into relatively large peptides [24]. Some of these peptides can bind directly to HLA-DQ2.5 or -DQ8 and trigger T-cell responses, which may result in local tissue damage [16,25]. In parallel, those gluten peptides can trigger the release of tissue transglutaminase 2 (TG2) which can deamidate some glutamine residues to glutamate in a large number of gluten peptides [16,26]. Deamidated gluten peptides can bind with much higher affinity to HLA-DQ2.5 or -DQ8, which can amplify the gluten-specific T-cell response. The activation of TG2 is thus a crucial step to increase the immunotoxicity of the gluten peptides [25]. The gluten-specific T-cell response induces the release of inflammatory cytokines that generate local inflammation [27]. 

## 3. Immune Activating Potential of Gluten Peptides

Hundreds of immunogenic peptides have been described in wheat, barley, rye and oats, which are able to activate T-cells [12,24,28]. However, not all gluten peptides are equally harmful to CD patients, as the number of epitopes present in these peptides may vary strongly between different cereals [29] and between cereal species [30]. Ciccocioppo et al. [31] suggested that gluten peptides are divided in two groups, toxic and immunogenic. Both pathways interact and potentiate each other to sustain the chronic process of the intestinal damage. Some peptides are capable of inducing non T-cell mediated mucosal damage when administered “*ex vivo*” on biopsies from celiac small intestine. Gluten Immunogenic Peptides (GIP) contain sequences which specifically stimulate T-cell lines isolated from peripheral blood of CD patients. Once a peptide is identified as immunogenic, it is considered relevant for those with CD, regardless whether or not it has also toxic properties [32]. The mechanism of toxicity for such non-*T* cell active peptide is not clear yet and there is no broad consensus about that classification of peptides. We will then refer in the rest of the manuscript to gluten toxic peptides to those with immunogenic activity.

The complete repertoire of peptides involved in the pathogenesis of CD remains a daunting task because of the great heterogeneity of gluten proteins [25,28,33,34,35,36,37,38]. Severalstudies have demonstrated that peptides derived from *α*-gliadins induce strong responses in the large majority of patients, while responses to the other peptides are less frequently found. It is possible that some immunogenic gluten epitopes may be tolerated by CD patients depending on the patients’ sensitivity to the different immunogenic epitopes [16,39,40]. In this scenario it becomes very important to accurately detect which immunogenic gluten epitopes are present in foods. 

Several gliadin peptides have been described to induce the adaptive immune response, but most of them are digested by gastric, pancreatic, and intestinal proteases. Two well-known main peptides remain undigested: the 33-mer (p55–87) and the 25-mer (p31–55). Consistently, these two peptides are active *in vivo* in the celiac intestine after gluten ingestion [41,42,43,44]. A peptide sequence located at position 31–43 of *α*-gliadin, which is contained in the 25-mer, represents a toxic peptide [45,46,47]. The p56–68 of *α*-gliadin, which is contained in the 33-mer, is considered to be the most important CD-immunogenic sequence within gluten [25]. The *α*-gliadin 33-mer (amino acid sequence LQLQPFPQPQLPYPQPQLPYPQPQLPYPQPQPF), contains three repetitions of the p56–68 of *α*-gliadin [44]. This peptide is present in the N-terminal repetitive region of *α*-gliadins and contains six overlapping copies of three different DQ2-restricted T-cell epitopes with highly stimulatory properties [44]. It harbors the p56–75 peptide (LQLQPFPQPQLPYPQPQLPY) that has been identified as the dominant gluten epitope [39,40]. The presence of the 33-mer in most common wheat and spelt cultivars have been recently detected and quantified by LC-MS/MS (Liquid chromatography tandem-mass spectrometry) ranging a level of 91–603 μg/g flour [48]. The special focus in the literature on this most immunodominant peptide seems to be justified.

Whereas the pathogenic role of CD4-mediated cells in CD is well-defined, several other studies have pinpointed the involvement of an innate, non-*T*-cell mediated immune response in intestinal mucosal atrophy [49]. Interestingly, among these peptides, two sequences of 18-mer and 25-mer lengths were truncated versions of the well-described 33-mer, and one peptide corresponded to the innate immune eliciting peptide 31–49 [34].

It is not fully understood how GIP can pass the epithelial barrier into the lamina propria to induce immune responses. Studies using biopsies from control and celiac patients found that p57–68 was completely degraded after mucosal to serosal transport. In contrast to biopsies of celiac patients, p57–68 was already partially degraded by brush-border peptidases in control subjects [50,51]. However, the use of intestinal biopsies from celiac and control patients to investigate the transport and degradation of GIP involve a complex interplay between various cell types and hardly allows to reveal the role the enterocytes in the metabolic fate of GIP and thus their role in antigen transport and presentation. The transport in the gut epithelium of two gluten peptides and among them, the immunogenic p56–68 peptide as representative GIP, has been studied [52]. GIP transport and increased uptake depended on the chain length as well as the integrity of the epithelial barrier system [52]. The absorption of gliadin peptides is complex. Reports indicate that GIP may be uptaken by the paracellular route [53]. Bruun et al. [54] also demonstrated that gluten fragments cross the intestinal barrier to be distributed to organs other than the gut, finding these gluten peptides in pancreas by mass spectroscopy and hypothesized the initiation of inflammation and induction of beta cell stress. The detection of gluten peptides in urine by using anti-gliadin 33-mer monoclonal antibodies (moAbs) demonstrated that gluten peptides can be absorbed, transported in the blood stream and excreted [55,56].

A systematic study of hundreds of gluten epitopes by ELISPOT revealed that three highly immunogenic peptides, derived from *α*-gliadin (ELQPFPQPELPYPQPQ), *ω*-gliadin/C-hordein (EQPFPQPEQPFPWQP), and *β*-hordein (EPEQPIPEQPQPYPQQ), account for 90% of the celiac-specific immuno-response elicited by the full proteome of wheat, barley and rye proteins [28,40,44,57]. Recently, the use of an HLA-DQ gluten tetramer test by flow cytometry comprising only five gluten epitopes (Table 1) could serve to identify celiac patients with 100% sensitivity and 90% specificity versus control with gluten containing diet. This test consists in recombinant and biotinylated HLA-DQ2.5 and HLA-DQ8 molecules covalently tethered with gluten peptides containing T-cells epitope sequences and conjugated with streptavidin. These DQ2–peptide complexes are stained for flow cytometry with mononuclear cells isolated from peripheral blood which allows CD4+ cells estimation [58,59]. Those results also suggested that a limited number of peptides represent more than 90% of the celiac specific T-cell response.

## 4. Analysis of Gluten Peptides

Many methods have been developed over the years for the detection of prolamins, including the polymerase chain reaction (PCR), LC-MS, and immunological methods based in antigluten peptide antibodies. Immunoassays as ELISAs (enzyme-linked immunosorbent assays) and LFDs (lateral flow devices) have been the method of choice because of combination of specificity, sensitivity, simplicity and cost effectiveness in the food industry to certify gluten-free food. An ideal antibody for gluten analysis should be not only a reliable indicator of the presence of prolamins from cereal species known to be toxic to CD patients but also should recognize the specific intramolecular regions responsible for such immunotoxicity [1].

The lack of an official reference material; the diversity of matrices, target analytes, sample extraction buffers, extraction time and temperature, as well as calibration standards; and differences in Ab specificity are factors contributing to the differences between the various methods of detection and quantification of gluten [64,65]. 

Several antibodies (Ab) have been raised against different prolamin epitopes (Table 2). Skerrit and Hill [66,67] developed a sandwich format that was approved as an official method by AOAC (Association of Official Agricultural Chemist) and it was used for many years in gluten analysis [1]. Other antibodies were raised against different epitopes of *α*-gliadin, such as PN3 (residues 31–49) for the toxic 19-mer peptides [46,68], CD5 (residues 51–75) or Abs against T-cell stimulatory peptides present in gluten [69]. The R5 moAb, raised against *Ω*-secalin from rye [70,71] recognizes highly repeated peptide sequences present in wheat, barley and rye grains. R5 reacts with high sensitivity against the epitope QQPFP as well as QLPFP, LQPFP, and QQQFP present in celiac-toxic sequences that occur repeatedly in gliadins, hordeins, and secalins [70]. However, when the reactivity to those epitopes has been brought to experimental testing, the sensitivity for some of the main immunogenic peptides, such as the gliadin 33-mer, was not significant [63,72,73]. The poor recognition of some of the main immunodominant gluten peptides may underestimate the immunogenicity of some foods, especially hydrolyzed food and beverages where GIP are more available [63].

Two moAbs, G12 and A1, were raised by the authors against the main immunogenic epitope of the *α*-gliadin 33-mer. These antibodies were able to detect the presence of gliadin 33-mer-related epitopes in prolamins from wheat, barley, rye and various oats varieties [60,74,77], as well as in food samples [62,63,78] and human samples to monitor GFD compliance and transgressions [55,61,79,80]. This technique has served to assess the efficacy of new experimental drugs or strategies to eliminate GIP during digestion [44,74,77,81]. 

Another anti 33-mer Abs, 14G11 and 13F6, have been recently used for the portable sensor device, Nima Sensor [73]. In addition to the affinity for the immunodominant gliadin 33-mer peptide not detected by R5, 14G11 and 13F6 affinity for gliadin also was 35 and 6.6-fold higher than the affinity of the R5, respectively. The performance of that device was tested in comparison with ELISA R5 and G12. Estimations in spiked samples with 10, 20, and 30 parts per million (ppm) of gluten in 13 different food matrices revealed that the device was able to detect 75.6%, 87.5%, and 93.6% positives, respectively. Intriguingly, the Codex Alimentarius Type I ELISA Sandwich R5 was underestimating gluten content in 13 out of 13 food samples with intended 20 ppm of gluten content in that study and 7 out of 13 for ELISA G12 [82].

## 5. Factors Affecting Gluten Content Estimations

Despite the lack of standardized reference material for gluten, there is a Codex Alimentarius Type I official method based on the ELISA R5 and Mendez Cocktail extraction since 2006 [83]. However, there are many food matrices difficult to measure due to their interferences with antibody binding, cross-reactivity, or problems in extracting the gluten [32]. In addition, there are open questions and wrong assumptions in assessing the gluten content:

### 5.1. Gliadin as Standard

The most typical calibration standard is gliadin because it is from wheat, which is the most frequent source of gluten; it is at least soluble in ethanol-water assuming that the proportion gliadin/glutenin in gluten is 1/1. In the formulation, the gluten content is calculated to be 2 times the estimated gliadin content. However, the proportion prolamin/glutenin varies greatly among the different cereals and cultivars [84]. A factor around 65:35 mainly for barley and rye has been described [1]. The underestimation or overestimation due to that assumption may be more frequent than initially assumed. 

### 5.2. Is Gluten Equivalent in Wheat, Barley, and Rye?

The reactivity and number of immunogenic peptide sequences may vary among different wheat [48,85,86] and barley [42] varietals. All wheat flours studied by Schalk et al [48] contained the 33-mer peptide. In contrast, the 33-mer was absent (< limit of detection) from tetra- and diploid species (durum wheat, emmer, einkorn), most likely because of the absence of the D-genome, which encodes *α*-gliadins. In Comino and coworkers [43], eight different barley cultivars were analyzed by ELISA G12 which revealed 25-fold differences in reactivity between the most and the least reactive barley cultivar. Three of those cultivars were analyzed by T-cell activation, and the hierarchy of immunogenicity with T-cells isolated from peripheral blood was consistent with the reactivity of the barley kernels. Therefore, the reactivity of the monoclonal antibodies used in the detection of gluten content may provide different estimations that should be verified with the real immunogenicity with human samples. 

### 5.3. Can Oats Contain Gluten?

Oats contains about 15% of avenin. However, this prolamin has not been broadly accepted to be named as gluten, in order to restrict the term ‘gluten’ to those prolamins generating clear food immunoreactivity to certain individuals. It has been assumed by many stakeholders that oats are naturally gluten-free and only when oats are contaminated with other immunotoxic cereals as wheat or barley, reactivity in gluten ELISA tests could be observed [87]. However, some pure oats cultivars have significant cross-reactivity with the most used monoclonal antibodies R5, G12 or Skerrit [60,88,89]. Some celiac T-cell activating sequences from oat have been identified [12,90,91] and some oat varieties have elicited early inflammatory events typical of CD [92]. Despite these evidences, it is still commonly believed that there is no reactivity in pure oats. However, most of the clinical studies where gluten challenges were made with non-reactive oats -assessed with the most commonly used immunomethods- suggest that oats may be safe for most celiac patients [91,93]. It remains to be verified whether 2 out of 15 patients with persistent histological deterioration after one year on oats challenge are due to lapses in the GFD or to the oat peptides [93]. In any case, it seems that lack of reactivity with immune assays (R5, G12, Skerrit, etc.) may guarantee the absence of gluten regardless of source-from oats itself or from wheat or barley contamination. 

### 5.4. How Much Immunogenic Gluten is There in Hydrolyzed Gluten?

Fragmented gluten proteins can be found in hydrolyzed food such as beers and baby food. The potential diversity in the generation of sequences, relative abundance and extension of the resultant gluten peptides is almost limitless [94]. The estimation of gluten equivalence in hydrolyzed gluten samples is thus a challenge. Firstly, peptides may have only one epitope per molecule. Therefore, the sandwich ELISA R5 underestimates the gluten peptide content of some beers [95]. This problem was solved by using competitive R5 ELISA that could measure single epitope-peptide. However, semi quantitative analysis of one hundred Belgium beers by a sandwich based A1/G12 lateral flow strips showed consistent results compared to a competitive G12 ELISA [62]. The most reactive fractions in High Performance Liquid Chromatography (HPLC) analysis for the G12-based immunomethods of samples of those beers also provided the highest immunoactivity. The G12 most reactive fractions also allowed to identify immunogenic gluten peptide sequences by Matrix assisted laser desorption/ionization-time of flight (MALDI TOF/TOF). Further analysis of one of the beers with differences in estimated gluten content by competitive R5 and G12, revealed that many of the identified immunogenic peptides showed tandem epitopes for A1/G12, explaining the consistency of the results with sandwich and competitive immunoassays [78]. The difference in estimations between different antibody-based method could be more appreciated in hydrolyzed food or beverages because of differential resistance of the corresponding epitopes observed [96]. Furthermore, the resistance of the gluten immunogenic peptides with G12 epitopes allowed to detect gluten in fecal and urine samples [55,61]. The amount of gluten in biological fluids was estimated by using a gliadin 33-mer calibrator, enabling the estimation of the mean daily gluten consumption in celiac patients [56].

**Figure 2:**
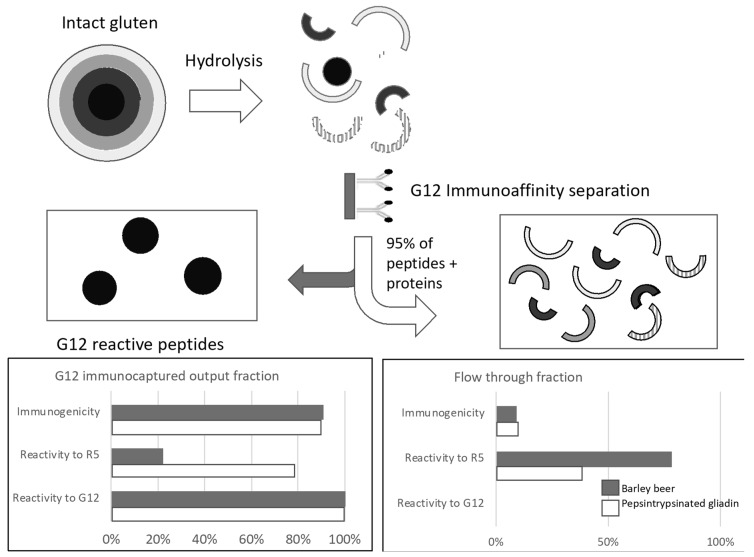
Diagram of the workflow for the selective capture of dominant celiac immunoactive peptides from hydrolyzed gluten. Semicircles with different colors represent peptides with epitopes not recognized by G12. Circular forms represent peptides with epitopes recognized by G12. Protein content by Bradford, T-cell reactivity (T-cell division and IFN-γ production), and G12 and R5 competitive ELISA were addressed to the different fractions.

The capacity of G12 Ab to immunocapture most of the T-cell activity of either a barley beer or a pepsin-trypsinized gliadin was also tested by using a G12-agarose resin for immunoaffinity separation of G12 binding polypeptides [63]. Less than 5% of the polypeptides of the beer or the hydrolyzed gliadin were retained in the G12 column, however, the captured peptides were able to generate about 90% of the immunogenicity estimated by celiac T-cell activation. Interestingly, 25% of the beer polypeptides and 65% of the gliadin hydrolysate that were able to be detected by R5 Ab were also retained in the G12 immunoaffinity resins. Those results indicated that most of the immunogenicity are detected by the G12 moAb and that the most used R5 Ab may underestimate the potential immunogenicity of certain hydrolytic material (Figure 2). 

### 5.5. How Much Gluten Does Genetically-Modified Low Immunogenic Cereal Contain? 

Biotechnological approaches, such as gene silencing by RNA interference (RNAi), have been used to produce low-gluten wheat in recent years. It was found that the silencing of γ-gliadin genes affects the rheological properties of wheat dough, where the downregulation resulted in dough that were stronger and more tolerant to overmixing [97,98]. As indicated in Table 3, many of the immunogenic gliadins were suppressed one to two orders of magnitude, with regards to the wild types, in a group of six modified plants by iRNA [99]. The reactivity for R5 epitopes decreased to less than 2% in some lines with regards to those of the wild types (from 1.227 ppm that of the transgenic plant to 64.395 ppm–114.043 ppm) using a competitive ELISA system. However, the glutenin content of such transgenic plants did not decrease significantly (75% to 143% of HMW glutenin with regards to the wild types), and neither did the protein level (13.1 to 15.7%). T-cell activation was tested with gluten extracts of the six transgenic plants. There was poor or no significant T-cell response in those six transgenic plants. Although immunogenic peptides have been described in HMW or LMW glutenins [24], those results with transgenic plants indicated that the immunoactivity of glutenins could be one order of magnitude lower than those of the gliadins. 

Recently, Sánchez-León et al. [100] have used the CRISPR/Cas9 technology to precisely and efficiently reduce the quantity of *α*-gliadins in the seed kernel, providing bread and durum wheat lines with reduced immunoreactivity for gluten-reactive consumers. ELISA tests with R5 and G12 moAbs showed a strong reduction in gluten content (up to 85%) in the modified lines compared to that of the wild type.

The first “ultra-low-gluten barley” was produced in Australia [104] using traditional breeding techniques, where three recessive alleles were combined to create low-hordein parental genotypes. The gluten content of barley variety was reduced to below 5 ppm estimated by ELISA. However, in that study the gluten content was not determined with an antibody method that recognized GIP.

### 5.6. Gluten Immunogenic Peptides Modified by Bacterial Enzymes 

The use of protease enzymes to inactivate gluten peptides has also been investigated as a method to remove the CD antigenicity of wheat and other cereals. The challenge lies in preventing the degradation of the protease enzymes in the gastrointestinal tract [105]. A pool of selected lactobacilli demonstrated the capacity of strongly hydrolyzed the wheat bread gluten (18,000 ppm) to less than 10 ppm after 360 min of treatment by R5 sandwich and competitive ELISA [106]. The specificity of G12 moAb to detect GIP was exploited to evaluate the immunogenic potential of the pool of peptides produced during bacterial colonization [107]. Wei et al. [96] found that exceptionally high gluten-degrading enzyme activities belonged to subtilisin protease family from *Rothia mucilaginosa* in human saliva could eliminate of the major gliadin epitope in preventing T-cell activation in the lamina propria and G12 ELISA assay was employed to monitor the epitope elimination. 

On the contrary, Janssen et al. [108] investigated the effectiveness of existing digestive enzyme supplements claimed to aid in gluten degradation by the R5 ELISA. Despite the observed reduction in the gluten content with the enzyme supplements by R5 method, authors argued that it did not reflect an actual breakdown of all immunogenic sequences because of being not enough specific for immunodominant peptide sequences from *α*-gliadins but is present in a number of *γ*-gliadin sequences. They corroborated by mass spectrometric analyses the gluten degradation products and certainly, the commercial enzyme supplements were completely ineffective in degrading immunogenic gluten fragments from both the *α*- and *γ*-gliadins. 

### 5.7. Gluten Immunogenic Peptide Can be Excreted in Stools and Urine

Despite the efforts to adhere to a gluten free diet, the treatment is very difficult because gluten is one of the most frequent ingredients in food. Most of the population have not such limitation and do not want to renounce to gluten containing food, therefore the coexistence with celiac population permanently maintains the risk. Methods to monitor gluten exposure in celiac patients are demanded either by clinical professionals and celiac patients or relatives. Recent transgressions to the gluten free diet could be determined by detection of gluten peptides in stools and in urine by A1 and G12 immunomethods [55,61,79,80]. The resistance of gliadin 33-mer to gastrointestinal digestion and the capacity to detect GIP after two days transit in the intestinal track were shown [61]. Western blot of gluten peptides from stools could not detect any defined band with G12 antibodies which demonstrated that most of the ingested gluten was hydrolyzed but not at the level to destroy the G12 epitopes [61]. Peptides with gluten epitopes for A1/G12 were also detected in urine of healthy and celiac volunteers with a lateral flow strip immunoassay and a lateral flow reader [55]. For detection in human samples, the content of GIP in either stools or urine were estimated with an gliadin 33-mer standard curve. Some of the conclusions from the analytical point of view about those works are the followings: (a) The sensitivity of the methods (0.16 mg 33-mer peptide/kg stools and 6 ng 33-mer peptide/mL urine) was sufficient to detect at least 97% of the population with expected daily gluten intake of 5–30 grams; (b) there were a very broad variation in the amount of excreted GIP (>100 fold differences between the GIP concentration among different volunteers ingesting similar amount of gluten) among different individuals ingesting a similar amount of gluten; and (c) there were no cross-reactivity with any of the samples of controlled volunteers showing. The first clinical studies with those methods concluded that 12–27% of celiac children and about 31.5% (female) to 60% (male) adult patients have ingested gluten last 2–4 days [55,109]. Those studies could also indicate very poor specificity and sensitivity of the most used methods for monitoring the adherence to the gluten free diet, either the dietary questionnaires or the serological markers, as the anti-tissue transglutaminase or anti-deamidated gliadin antibodies. The introduction of GIP as an assessment tool of GFD adherence may reduce the need for endoscopy.

Despite the large differences in the GIP concentrations among individuals, there was significant reduction in the mean GIP content of those celiac individuals with detectable GIP in stools compared to those ones with no restriction in the diet. The GIP concentrations in stools and in urine after gluten intake of define amount of gluten intake were used to estimate the average gluten intake in celiac disease [56]. The mean daily gluten consumption of celiac volunteers under a gluten free diet was estimated to be between 0.2 to 0.4 g of gluten, and 6–8 g for control volunteers with no restriction diet. Additional estimations with the analyses of the latiglutenase data for CD individuals with moderate to severe symptoms indicated that patients ingested significantly more than 200 mg/day of gluten [56]. The GIP content estimated with gliadin 33-mer peptide standard and anti 33-mer antibodies could be useful to estimate the immunogenic gluten exposure in celiac patients.

## 6. Conclusions and Future Directions

After decades of gluten analysis by immune methods, there is no consensus reference material for gluten. The definition of gluten is still an unsolved issue. The amount of cereal varieties, proteins, peptide sequences, hydrolytic and other industrial processes, as well as the diverse individual response to gluten peptides, make the detailed analysis of the potential risk of food stuff extremely complex. However, it is reasonable to propose that the concept should be linked to the immunogenic activity in celiac patients. In the last years, the advance in the scientific knowledge and pathology of CD has allowed to identify not only gluten peptide sequences that trigger the immunological response by T-cell activations in celiac patients, but also the most immunodominant among them. There are already immune methods with reported characterization of the consistency with “*ex vivo*” quantified immunogenicity with T-cells from celiac individuals. A limited set of dominant GIP has already been characterized as responsive of most T-cell response in CD. GIP could be detectable in very different environments including hydrolyzed food and beverages, excreted in stool and urine, what is consistent with the resistance to digestion of GIP and the generation of systemic symptoms of gluten-reactive populations. A limited number of GIP could be obtained by synthesis at high purity or quantity, and could be used as calibrator of either immune methods or mass spectrometric analysis. Those relevant gluten peptides may be considered as future reference materials to determine risky gluten exposure, before and after consumption.

## 7. Patents

The following patents are part of this work: Determination of levels of immunogenic gluten peptides in human samples (WO2012089868) and Detecting gluten peptides in human fluids (WO2016005643).

## Figures and Tables

**Table 1:** Most relevant Gluten Immunogenic Peptides (GIP).

Peptide Sequence	Origin, Description and Practical Evidences	References
LQLQPFP(QPQLPYP)_3_QPQPF	*α*-gliadin 33-mer. Correlation between food immunoreactivity with T-cells and reactivity of anti-33-mer antibodies among different oat and barley cultivars, and beer fractions. Suppression of 90% immunogenicity with G12 anti-33-mer antibody gluten peptides	[12,43,60,61,62,63]
EPEQPIPEQPQPYPQQ	*α*-gliadin	[57]
EQPFPQPEQPFPWQP	*ω*-gliadin/C-hordein	[57]
ELQPFPQPELPYPQPQ	*β*-hordein	[28]
QLQPFPQPELPY	DQ2.5-glia-*α*1a. Use in HLA-DQ Gluten tetramer tests	[59]
PQPELPYPQPE	*β*-hordein. DQ2.5-glia-*α*2. Use in HLA-DQ Gluten tetramer tests	[28,59]
QQPFPQPEQPFP	DQ2.5-glia-ω1. Use in HLA-DQ Gluten tetramer tests	[59]
EQPFPQPEQPFPWQP	*ω*-gliadin/C-hordein. DQ2.5-glia-*α*2. Use in HLA-DQ Gluten tetramer tests	[28,59]
EPEQPIPEQPQPYPQ	*α*-gliadin. DQ2.5-hor-3. Use in HLA-DQ Gluten tetramer tests	[28,59]

**Table 2:** Commercial antibodies used to detect gluten and preferably recognized cereals and epitopes.

Monoclonal Antibodies	Prefered Epitope	References
G12	QPQ-(L/Q)-P-(Y/F)	[74]
A1	Q-(Q/L)-P-(Y/F)-PQP	[74]
R5	QQPFP	[70]
401.21	PQ-(PQ/QP)-PFP-(QE/EES)	[75]
α20	F-RPQQPYP-Q	[76]
14G11	*α*-gliadin 33-mer, ND	[73]
13F6	*α*-gliadin 33-mer, ND	[73]

ND, not determined the minimal epitope. Red and black colors indicate different amino acid combination, respectively. Green color indicates four different amino acid combination epitopes.

**Table 3:** Prolamin and glutelin content of cereals and their immunoreactivity in vitro.

Cereal	Prolamin (%)	Glutelin (%)	Immunoreactivity ^*α*^	Detection Method	References
Wheat	5.94 ^a^	2.98 ^a^	++	G12 and A1 moAbs	[77,79,80]
Transgenic wheat	1.96 ^b^	4.16 ^b^	ND. 85% G12 reactivity reduction	R5 and G12 moAbs	[100,101]
Barley	3.13 ^a^	1.1 ^a^	++ *^β^*	G12 moAb	[43,74,78]
Rye	2.53 ^a^	0.55 ^a^	ND	G12 and A1 moAbs	[74,77]
Oats	1.29 ^a^	1.01 ^a^	+/− *^β^*	G12 moAb	[60,74,77,78
Rice	0.54 ^c^	6.66 ^c^	−	G12 moAb	[60,74,77,78]

*α* PBMCs proliferation and IFN-*γ* response. (++) Gliadin-like, (+) Less Gliadin-like, (+/−) More Orzein-like, (-) Orzein-like. *β* Differences among varieties. (a) Osborne method [102] (b) Kjeldahl method [101] (c) SDS-Page [103]. ND, Not determined. (moAbs) monoclonal antibodies.
